# Age Differences in Long Term Outcomes of Coronary Patients Treated with Drug Eluting Stents at a Tertiary Medical Center

**DOI:** 10.1155/2013/471026

**Published:** 2013-06-02

**Authors:** Nicolas W. Shammas, Gail A. Shammas, Peter Sharis, Michael Jerin

**Affiliations:** Midwest Cardiovascular Research Foundation, 1236 E Rusholme, Suite 300, Davenport, IA 52803, USA

## Abstract

We evaluate differences in outcomes in younger (<65 years) and older (≥65 years) patients for target lesion failure (TLF) at 2-year follow-up in an unselected consecutive series of patients treated with the everolimus- (EES) and paclitaxel-eluting (PES) stents at a tertiary medical center. 348 consecutive patients (younger 150; older 198) stented with the EES and PES were retrospectively analyzed. The primary endpoint was TLF (composite endpoint of cardiac death, non fatal myocardial infarction due to index vessel and target lesion revascularization (TLR)). At 2 years follow up, younger versus older patients had the following outcomes respectively: TLF 27.7% versus 25.5% (*P* = 0.71), TLR 24.8% versus 21.4% (*P* = 0.52), cardiac death 3.4% versus 2.5% (*P* = 0.75) and definite and probable stent thrombosis (2.0% versus 1.0%). Multivariate analysis showed that renal failure (odds ratio: 2.55, *P* = 0.045), number of stents per patient (odds ratio: 1.60, *P* = 0.001) and younger age (odds ratio: 0.97; *P* = 0.010), but not gender, diabetes or type of DES stent (EES versus PES) predicted TLF. We conclude that older age was not a predictor of TLF at 2-year follow-up after adjusting for renal insufficiency, number of stents used per patient, gender, diabetes and type of DES used.

## 1. Introduction

There is a relative paucity of data on clinical outcomes of elderly patients undergoing contemporary percutaneous coronary interventions (PCIs) with drug eluting stents (DESs). Elderly patients have been frequently excluded from randomized clinical trials because of multiple comorbidities making available data on DES difficult to generalize to older patients [1]. Several reports indicated that older age (>65 years old and particularly >75 years old) is an independent predictor of adverse events after percutaneous coronary interventions (PCIs) with higher inhospital vascular complications and cardiac death [2, 3] and higher long term cardiac mortality [4]. Other reports suggested that older age, after adjusting for various clinical and procedural variables, does not appear to independently predict adverse early or late outcomes after PCI [5, 6].

The paclitaxel-eluting stent (PES) Taxus Liberte (Boston Scientific, Natick, MA, USA) and the everolimus-eluting stent (EES) Xience (or Promus) (Abbott Laboratories, Abbott Park, IL, USA) are drug eluting stents (DESs) that showed lower TVR than bare metal stents (BMSs) irrespective of age, although higher mortality and myocardial infarction seem to increase with higher age groups [7]. 

In this report, we examine our own data for differences between younger (<65 years) and older (≥65 years) patients in target lesion failure (TLF) in an unselected consecutive series of patients treated with the EES and PES stents and followed for at least 2 years at our medical center. 

## 2. Methods

Unselected consecutive patients treated with the PES and EES stents were retrospectively recruited from a single center. Patients who received mixed stents during the index procedure or bypass graft treatment were excluded. The choice of the drug eluting stent was left to an individual operator. Demographics, clinical, procedural, and angiographic variables are shown in Tables 1 and 2. Angiographic variables (including ejection fraction from left ventriculography) were obtained by an independent blinded review of the angiograms to patients' clinical variables and outcomes. 

Followup was completed at 2 years from the index procedure using medical records, phone calls, or both. The protocol was approved by our Institutional Review Board. Patients were mailed a brief letter describing the protocol, followed by a phone call to obtain verbal consent to be part of the study. Patients who died during the follow-up period had their death certificates retrieved to verify the cause of death. 

 The primary outcome of the study was differences in target lesion failure (TLF) defined as cardiac death, nonfatal myocardial infarction related to target vessel and target lesion revascularization (TLR) between younger (<65 years) and older patients (≥65 years) patients. Differences in secondary outcomes (target vessel revascularization (TVR), target vessel failure (TVF), acute stent thrombosis (ST) as defined by the Academic Research Consortium (ARC) [8], nonfatal MI, and cardiac death) were prespecified as secondary endpoints. 

### 2.1. Statistical Analysis

 Descriptive analysis was performed on all variables. *t*-testing was used for continuous variables and chi-square testing for dichotomous variables. Univariate analysis compared the demographic, clinical, angiographic, and outcome variables between males and females. TLF survival analysis (Kaplan-Meier) was performed for both subgroups. Logistic regression analysis modeling for age, gender, obesity, diabetes, hypertension, LM lesions, renal insufficiency, number of stents per patient, and stent type was performed. SPSS (IBM, NY, USA) software was used to conduct the analysis. 

## 3. Results

 Descriptive and clinical variables are shown in Table 1. There were more males, smokers, obese patients, and a higher incidence of premature family history of coronary disease in the younger group. Older patients had more peripheral vascular disease and hypertension. The majority of both younger and older patients were still on dual antiplatelet agents on followup.

Procedural and angiographic variables are shown in Table 2. Older patients had a nonsignificant trend for more left main and more vessels treated per patient, but otherwise angiographic variables appear to be similar between the 2 groups. Both younger and older patients received equal proportion of EES and PES stents. There was a high prevalence of treated bifurcating, left main, and ostial disease in both groups. 

 The reasons for the index angiogram are listed in Table 3 and appear to be similar in both younger and older patients. The majority of patients were symptomatic with either acute coronary syndromes or chest pain with abnormal functional testing. A smaller percentage similar between the 2 groups had no symptoms and were treated for an abnormal functional test or as part of a staged procedure. Despite the complex disease treated, angiographic success, defined as obtaining a residual stenosis of less than 30%, was achieved in all cases. 

 At 2-year followup, the primary endpoint of TLF was 27.7% and 25.5% in both younger and older cohorts, respectively, (*P* = 0.711) (Table 4) with no statistical difference in the secondary endpoints between TVF (36.0% versus 32.8%, *P* = 0.569), TLR (24.8% versus 21.4%, *P* = 0.518), cardiac death (3.4% versus 2.5%, *P* = 0.750), definite and probable stent thrombosis (2.0% versus 1.0%), and nonfatal myocardial infarction (4.0% versus 4.5%, *P* = 0.475). 

Logistic regression analyses showed that renal failure (*P* = 0.045, odds ratio: 2.552, 95% confidence interval: 1.021 to 6.381), number of stents per patient (*P* = 0.001; odds ratio: 1.603, 95% confidence interval: 1.255 to 2.047) and to a lesser extent younger age (*P* = 0.010; odds ratio: 0.969, 95% confidence interval: 0.947 to 0.992), but not gender, diabetes, or type of DES stent (EES versus PES) predicted TLF.

## 4. Discussion

Several reports indicated that older age is an independent predictor of adverse events after PCI with higher inhospital vascular complications and cardiac death [2, 3] and higher long term cardiac mortality [4]. Feldman et al. [3] reported the outcomes of PCI patients in 3 age groups (<60, 60–80, and >80 years) from the New State Angioplasty Registry. Older patients had more comorbidities with more extensive coronary disease, hypertension, peripheral vascular disease, and renal insufficiency. In the emergency PCI group, there was an age-related incremental increase in inhospital mortality (1.0% versus 4.1% versus 11.5%, *P* < 0.05) and major adverse events (1.6% versus 5.2% versus 13.1%, *P* < 0.05). Similarly, in those patients presenting for elective PCI group, there was also an age-related incremental increase in mortality (0.1% versus 0.4% versus 1.1%, *P* < 0.05), and adverse events (0.4% versus 0.7% versus 1.6%, *P* < 0.05) but to a lesser extent than emergency patients. In their series, multivariate analysis demonstrated that age was strongly predictive of inhospital mortality for all PCI patients. In addition, Assali et al. [2] compared the outcomes of 266 consecutive patients ≥75 years with 1681 consecutive patients <75 years of age undergoing nonemergent PCI. Elderly patients had a higher length of hospital stay, more vascular complications, and bleeding complications. Multivariate analysis demonstrated that age ≥75 years was found to be an independent predictor of inhospital cardiac death (odds ratio = 3.9; 95% CI = 1.3–11.5; *P* = 0.015). 

In our reports, cardiac mortality and TLR were not different between younger and older patients at 2-year followup. There was a statistical increase in the odds ratio of TLR for younger patients, although the magnitude of this change was clinically insignificant. This is similar to what Costa Jr. et al. [4] reported in 1364 patients undergoing PCI and stratified by age. In their cohort, and despite an increase in cardiac mortality in the elderly, the long term adverse events were similar between younger and older patients (>75 years). Similarly, Xu et al. [5] reported in 333 patients undergoing PCI that there were no differences in restenosis, TLR, stent thrombosis, bleeding, and stroke in younger versus older patients on followup at 7 months. Finally, Rathore et al. reported no differences in procedural success, inhospital adverse cardiac events, and major adverse events at 1-year between younger (<65 years) and older (≥65 years) women undergoing PCI. In fact, similar to our data, older patients had less likely ischemia-driven revascularization at 1-year followup. Although we see a statistical reduction in the odds for TLF in older patients in our cohort, the magnitude of the change is small and likely to be clinically insignificant. Although elderly patients are more likely to have a higher cardiac mortality with long term followup, particularly when followup starts at an advanced age (>75 years or 80 years of age), there is no apparent disadvantage in revascularization of elderly patients compared to younger ones with respect to recurrent revascularization and TLF. This data supports the conclusion that elderly patients need to be given the option of revascularization when feasible with expected TLF similar to the younger population. 

One of the limitations of this study is its relatively small size and its retrospective design. The difference in TLF rates between the younger and older populations is, however, very small (2.2%) and unlikely to be significant even with a much larger number of patients enrolled. Also, bias has been reduced by an unselected cohort of patients, and the data appears consistent with published outcomes in younger versus older patients in the current era of post-PCI. Finally, we selected ≥65 years of age (rather than ≥75 years) to be the cutoff limit for defining an “older” population. Whether our data can be extrapolated exclusively to patients over 75 years of age is unknown. 

## Figures and Tables

**Table 1:** Demographic and clinical variables.

	<65 years	≥65 years	*P* value
Males (%)	69.3	59.1	0.056
Body mass index	33.1 ± 7.2	29.0 ± 5.7	0.001
NY heart class for failure on presentation (%)			NA
Class 0: none noted	83.2	78.2	
Class I: dyspnea with high activity	4.7	8.6	
Class II: dyspnea with regular activity	10.1	9.1	
Class III: dyspnea with minimal activity	1.3	2.5	
Class IV: dyspnea at Rest	0.7	1.5	
Prior percutaneous coronary intervention (%)	70.5	70.2	1.000
Prior coronary artery bypass surgery (%)	18.7	25.3	0.155
Previous myocardial infarction (%)	37.3	35.4	0.736
Family history of premature CAD (%)	58.5	27.7	0.001
Renal failure (creatinine ≥ 2.0 at baseline) (%)	5.4	9.7	0.159
Chronic lung disease (%)	11.3	16.7	0.169
Peripheral vascular disease (%)	8	17.2	0.016
Hypertension (%)	74	87.9	0.001
Cerebrovascular disease (%)	2.7	4.6	0.407
Hyperlipidemia (%)	86	88.4	0.519
Smoking history (%)	69.3	58.4	0.043
Diabetes mellitus (%)	38.3	36.4	0.737
Clopidogrel on followup (%)	86.7	83.5	0.515
Aspirin on followup (%)	96.9	94.1	0.408

NS: nonsignificant, CAD: coronary artery disease, and NA: not applicable.

**Table 2:** Procedural and angiographic variables.

	<65 years	≥65 years	*P* value
	*n* = 150	*n* = 198	
Vessels treated (*n*)	249	385	
Vessels treated per patient	1.7	1.9	
Mean number of stents per vessel (*n*)	2.1 ± 1.4	1.9 ± 1.2	0.073
Stent type per patients			0.449
Everolimus	56.7	52.5	
Paclitaxel	43.3	47.5	
Lesion location (%) per patient			
Ostial lesion	20.0	20.7	0.803
Bifurcating non left main	59.6	55	0.388
Left main	13.3	25.2	0.360
Ejection fraction (%)	51 ± 13	50 ± 16	0.767
Vessels with restenotic lesions (%)	28.2	23.0	0.157
Lesion length per patient treated (mm)	66.8 ± 54.8	67.9 ± 50.2	0.832
Diameter of index vessel (mm)	3.0 ± 0.6	3.0 ± 0.50	0.993

**Table 3:** Reasons for the index angiogram.

Percentage	<65 years	≥6*5 *years	*P* value
	*n* = 150	*n* = 198	NA
Unstable Angina	50.6	45.9	
STEMI	3	3.6	
Chest pain with abnormal functional test	16.3	12.7	
Abnormal functional test, asymptomatic	5.4	6.8	
Staged procedure	22.3	25.9	
Unexplained dyspnea	0	1.8	
Cardiomyopathy	0.8	0.9	
Others	1.6	2.4	

NA: non applicable.

**Table 4:** Two-year primary and secondary outcomes.

	<65 years	≥65 years	*P* value
	*n* = 150	*n* = 198	
Target lesion failure (%)	27.7	25.5	0.711
Target vessel failure (%)	36	32.8	0.569
Target lesion revascularization (%)	24.8	21.4	0.518
Target vessel revascularization (%)	33.1	28.8	0.411
Stent thrombosis			NA
None	95.9	97.4	
Definite	2	0.5	
Probable	0	0.5	
Possible	2	1.5	
Nonfatal myocardial infarction (%)	4.0	4.5	0.475
Cardiac death (%)	3.4	2.5	0.750

NA: non applicable.
